# Resettlement experiences and resilience in refugee youth in Perth, Western Australia

**DOI:** 10.1186/s13104-015-1208-7

**Published:** 2015-06-10

**Authors:** Jaya Earnest, Ruth Mansi, Sara Bayati, Joel Anthony Earnest, Sandra C Thompson

**Affiliations:** International Health Programme, School of Nursing and Midwifery, Curtin University, GPO Box U1987, Perth, WA 6845 Australia; Combined Universities Centre for Rural Health, University of Western Australia, 167 Fitzgerald St, Geraldton, WA 6530 Australia

**Keywords:** Refugee youth, Coping strategies, Resettlement, Resilience, Western Australia

## Abstract

**Background:**

In Australia, the two major pathways of refugee entry are the United Nations High Commissioner for Refugees resettlement programme and irregular maritime arrivals (IMAs) seeking asylum. The Australian Government’s policies towards IMAs since July 2013 are controversial, uncompromising and consistently harsh, with asylum seekers held in detention centres for prolonged periods. Refugees and asylum seekers have distinct and unique stressors that make resettlement difficult.

**Methods:**

This exploratory study examines resettlement experiences for refugee youth in Western Australia using the psychosocial conceptual framework and qualitative methods. Focus group discussions and key informant interviews were undertaken with verbatim transcripts analysed using thematic analysis to identify themes.

**Results:**

Themes documented that language and its impact, and experience with education, health, and social activities, support structures provided to youth and supporting future aspirations as critical to successful resettlement. This exploratory study contributes to developing a broader understanding of the resettlement experiences of refugee youth, drawing on their current and past experiences, cultural differences and mechanisms for coping.

**Conclusion:**

Fluency in English language, especially spoken, was a facilitator of successful resettlement. Our results align with previous studies documenting that support programs are vital for successful resettlement. Although faced with immense difficulties refugee youth are resilient, want to succeed and have aspirations for the future. Strategies and recommendations suggested by refugee youth themselves could be used for developing interventions to assist successful resettlement.

## Background

As a result of conflict, persecution and wars in other countries, each year Australia receives many requests for asylum and humanitarian resettlement. According to the Australian Department of Immigration and Citizenship (DIAC), in 2011–2012 almost 43,000 applications were lodged for protection visas [1], of which 7,038 were granted [2]. Half (51%) of the applications were from irregular maritime arrivals (IMAs)—those who arrive by boat and the remainder were lodged through the United Nations High Commissioner for Refugees (UNHCR) [3, 4]. Globally, 46% of the refugee population is aged under 18 years [3] and in Australia, for the 2012–2013 program year, the largest proportion of the humanitarian visa applications (those seeking asylum or refugee resettlement) were in the age group of 15–19 years [1]. Children and young people seeking asylum, are particularly vulnerable to the long-term impact of human rights violations, discrimination and trauma experienced in their home country, transition and resettlement country [5]. Given the importance of adolescence and early adulthood as a critical time of significant physical, mental and emotional development, support programmes for the successful resettlement of refugee youth should be a priority for host countries.

### Refugee support

Refugees who cannot go back to their country due to persecution are resettled by the United Nations High Commissioner for Refugees. Most of these refugees are resettled in the USA, Canada, Australia, some European, Nordic and Latin American countries [6]. Australia is a signatory to Geneva Convention and has granted 75,000 humanitarian visas since World War II in its humanitarian entry program [7]. Australia continually reviews its resettlement programmes [settling in payments (funds provided when refugees first come into the country, community support, language, literacy and numeracy classes, guidance in job seeking) reflecting its commitment to helping humanitarian entrants into Australia [5, 8–10]. After resettlement, the first 3–6 months are funded by DIAC through the International Humanitarian Support Scheme [11]. Those arriving through the overseas resettlement route receive continued support through Medicare (the Australian government funded healthcare system) and Centrelink (the Australian government funded social security payment agency) [11]. In recent times, DIAC’s emphasis on self-reliance has led to a reduction in support especially of social services and sponsored housing. Policy changes often create confusion, a lack of clarity regarding available services and increase pressure on community and volunteer organisations [12].

### Factors affecting resettlement

An Australian study reported on psychological factors that facilitates refugees’ resettlement experience [13]. Overall wellbeing was linked to ‘indicators of belonging’, specifically social status, support, lack of discrimination and a peaceful environment. The study documented that if these indicators are addressed then refugee youth felt included in society and would thrive [13]. Asylum seekers and refugees arrive in Australia with a background of human rights violations, often presenting complex health issues such as torture, exposure to prolonged trauma and mental health issues [5, 14, 15]. In addition, separation from family often led to feelings of loneliness, guilt and ostracism [14, 15]. Community based organisations provide limited services, but the absence of essential ongoing mental healthcare impacts the overall wellbeing of refugees [11, 12].

Refugees are often stigmatised and discriminated against, and this discrimination often increase with their inability to understand the language and culture [5]. In a study of Afghan female youth in Melbourne, resettlement was improved through relationships with others from the same cultural background. The same study found that those attending English classes for over 6 months felt more optimistic and competent in their ability to communicate [5]. Refugee youth also recognise and value the freedom that they have in Australia but are often confronted with cultural conflict [5].

### Resilience

There is a direct link between resilience and coping strategies in the context of the vulnerable populations [16]. Luthar and Cicchetti ([16], p 2) refer to resilience as ‘positive adaptation despite adversity’. Programs which focus on integrated and comprehensive interventions by working with the individual, family unit and community have been successful in increasing resilience within marginalised societies, and have shown that emotional distress in the absence of strong social structures impacts negatively on resilience [16–18]. Another study documented that an individual’s resilience was enhanced if there was a sense of hope, strong structures and leaders, social support and a feeling of personal security [19, 20].

Research to ascertain the resettlement experiences of refugee youth is needed, particularly studies which focus on the development of intervention models that address the complex needs of this population and build resilience [18]. This cross-sectional study undertaken in Western Australia hoped to address this gap and was framed in the context of Australian humanitarian policies.

## Methods

Due to the sensitive nature of the issues being explored—resettlement issues faced by refugee youth, a qualitative approach was considered appropriate as it allows for an in-depth exploration of issues. The study comprised of focus group discussions (FGDs), key informant interview and a systematic review of literature. This study was conducted between July and November 2013, as part of a larger project and examined the resettlement and coping strategies amongst refugee youth from Afghanistan, the Democratic Republic of Congo (DRC), Ethiopia, Sudan, South Sudan, Iraq, Pakistan and Burma at an Intensive English Centre (affiliated to a secondary school that offers English language instruction to young people aged between 16 and 20 years) and Centacare Employment and Training (CET: a registered training organisation providing language, literacy and numeracy skills) in Western Australia (WA). Purposive sampling was used to recruit participants, the institutions were approached to take part in the study as they were known among WA migrant networks and had made a difference to the community and also played a role in empowering refugee youth.

The study examined resettlement experiences and social and community support available to the refugee youth in regards to health, education, social support and employment in WA. It also explored the participants’ coping strategies as they established a new life in WA.

### Conceptual framework used for the study

**Figure 1:**
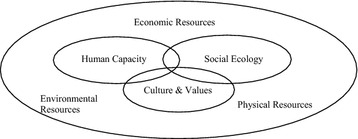
The domains of the psychosocial framework [21].

The study was underpinned by the psychosocial conceptual framework which rests on the assumption that the psychosocial well-being of an individual is defined by three core domains: human capacity, social ecology and culture and values. These domains map the **human** (physical and mental health and well-being, the skills and knowledge of people, and their livelihoods), **social** (relations within families, links with peer groups, religious, cultural civic and political institutions) and **cultural** (cultural values, beliefs, practices, human rights) **capital** available to people responding to challenges of prevailing events [21]. Broad themes raised for discussion and drawn from the analysis explored factors that enhance resilience; the socio-cultural contexts of the adolescents’ lives; their sense of belonging and community cohesion experienced as they resettled in Australia; and their perceptions regarding future aspirations. Figure 1 illustrates the interlocking of these domains: human capacity, social ecology and culture and values. These domains also map the human social and cultural capital available to people responding to the challenges of prevailing events and conditions. These domains have utility and validity as discrete ‘lenses’ through which the impact on resources at the community level is considered [21].

### Participant recruitment and data collection

Participants were selected using opportunistic and purposive methods, to ensure selection of informants who were uniquely positioned to share their experiences of the process of resettlement. Refugee youth were recruited at the Intensive English Centre, Cyril Jackson Senior College and Centacare Employment and Training in WA. Three FGDs were conducted, one with one male and two female participants from the DRC, the second with four male participants from Afghanistan, and the third group consisted of four females and four males, two from Burma, two from Iraq, two from South Sudan, one Ethiopian and one Pakistani. Almost all the participants were adequately proficient in English to be able to converse and share their thoughts except for two male youth in the third group who had a lower level of participation in the FGD. In the FGDs, participants shared demographic information, their mode of migration, languages spoken, their experiences of health and education services, social support, acculturation, and aspirations for the future.

The FGDs were conducted such that refugee youth from the same cultural background were interviewed in the same group so that they felt at ease except for one of the FGDs, which was organised such that participants with the same level of English participated in the interview. There were no gender based questions so males and females participants were interviewed at the same time. The first two FGDs were conducted at the Cyril Jackson School campus and the third one at the Centacare in mid-2013. Two research assistants from ethnic minority backgrounds and the second author conducted the focus groups. The FGD was concluded when data saturation was achieved and no new information was forthcoming from the groups. Every effort was made by the research team to avoid bias.

After the FGDs, two key informants (KIs) were purposively recruited from amongst refugee advocates with knowledge of the diverse experiences of asylum seeker and refugee youth and the complex issues surrounding resettlement in Australia. The first KI was a migration support officer with a social work background working with the West Australian Red Cross. The second KI worked as a human rights lawyer with refugee support centres for more than 5 years. Both KIs were passionate about refugee and asylum seeker resettlement issues. Semi-structured, in-depth interviews were conducted with KIs and the issues discussed were coping and resilience during resettlement, humanitarian resettlement quotas and the process of on-shore and off-shore asylum applications. Their responses supported the responses of the FGD participants.

### Ethics approval

Ethics approval was obtained from Curtin University’s Human Research Ethics Committee. Written informed consent was obtained from all participants. Under aged participants (under 18 years) obtained consent from their parents or legal guardians and also signed the consent forms. Information sheets were distributed prior to the study and participants were informed that they could withdraw from the study at any time.

### Research rigour

This research used dependability, credibility, confirmability and transferability [22], important components of this qualitative research [23]. Trustworthiness was established through reflexivity and clearly defining the roles of the researchers and refugee youth in every stage of data collection and findings [24]. Member checks with key informants, clarified issues that emerged during data analysis to enhance validity [22]. An audit trail [24], ensured that methods were documented so that data analysis and results were confirmed and transferable and the study replicated by other researchers.

### Data analysis

The psychosocial framework allowed for recurring and significant themes to be identified under the themes of human capacity, culture and values [21, 25]. The FGD and KI interviews were tape-recorded and transcribed verbatim for analysis. A two fold process to data management and analysis using a modified framework approach was adopted through the reading and re-reading of the transcriptions to identify themes. This allowed the researchers to review the data, to generate themes, identify quotes to enhance rigour [26, 27]. Member checking was undertaken through interpretation of themes between members of the research team, themes were shared informally with some participants and key informants to corroborate themes and validate conclusions.

The second stage refined the existing themes [28, 29] through a process of reviewing, grouping and identifying overlapping themes and identifying key issues that were recurring. The key emerging themes were discussed amongst the researchers and the responses of youth participants triangulated with findings from the two key informant interviews. Themes drawn from the analysis and underpinned by the psychosocial framework are presented in Table 1. The theme ‘aspirations for the future’ overlapped all domains and has thus been included in all the domains in Table 1. Participant quotes relevant to each themes were incorporated into the analysis to validate the themes and were selected by a member of the research team who had undertaken the interviews and then were considered by other members of the research team, finally the third author went reviewed the themes and quotes.

**Table 1:** Table of themes drawn from the analysis

Social ecology	Social activities
	Support structures
	Religion
	Aspirations for the future
Human capacity	Language
	Education
	Health (both mental and physical)
	Employment
	Aspirations for the future
Cultural capacity	Connection to family
	Loss of family member left behind
	Family roles
	Parental language and work skills
	Aspirations for the future

## Results

### Study participants

The participants from the DRC were all aged 19 years and had been in Australia from 3 months to 1.5 years on humanitarian resettlement visas. Two had lived in Tanzania, and one in Zimbabwe. They had come with their families and spoke Swahili, French, Shona and English. Three had high school education and were attending Intensive English classes over 16 weeks before moving to mainstream classrooms. Four participants from Afghanistan were male, one was aged 17, two were 18, and one was 20 years old. Three participants had arrived in Australia alone by boat and sought asylum, 12–18 months previously. They spoke Hazaragi, Dari, Persian and English. Participants in the third group were aged 19–28 years and entered Australia on Humanitarian resettlement visas except one IMA. Four were with families upon arrival, while the rest of the group entered Australia alone, the time being resettled in Australia ranged from 1 year to 8 years with most of the participants being here <2 years.

### Human capacity themes

#### Language

Fluency in English language, especially spoken, was either a barrier or facilitator to successful resettlement. Only one Congolese participant had spoken English prior to coming to Australia, although she was not proficient in the language. The youth reported that if they felt confident with the language, they had less problems overall and settled in faster.

*“Yes it is the most difficult the language. You can’t talk to anyone, everywhere you go. Even at home when someone is calling from company, Centrelink or hospital you cannot communicate with them. I just tell them sorry I can’t speak English. But after that I feel very sad.”*—***Congolese Female***

*“When I came here (to Australia) I didn’t want to do English because honestly I was ok with English but the school principle told me I don’t have any papers showing that. Sometimes I feel like I have lost so much time doing English when I was supposed to go to mainstream classes.”*—***Congolese Female***

#### Education

All the youth had some formal education in their countries of origin, however, none of them were taught English. The youth attributed a positive educational experience in the intensive language program to the teacher’s helpfulness and friendliness, a smaller classroom size and teachers who were able to speak their native language. Going back to school or missing a year due to the lack of recognition by the Australian education system of prior education was reported as a problem. Six of the seven participants did not have proper documentation to prove the level of schooling they had achieved previously. In addition to repeating school, KI 2 mentioned the problem of age limit for school enrolment. Many refugee youth have spent years in refugee camps and detention centres without any schooling and have interrupted schooling however they cannot be enrolled in schools once they turn 18 years and have to go to an adult education centre.

*“It’s good because we’ve got a teacher and they help us. And they help us with different languages, and my math teacher speaks Swahili and another teacher she speaks a bit French because she was in France. It helped me when I first came to Australia”*—***Congolese Male***

*Even the teacher when he speaks to the students he respects the student. They are the small things. The respect is good. You feel good that the teacher respects you and try to make you happy. I want to study more and more.”*—***Iraqi Female***

*Many youth are missing out on schooling, whether it’s because they are in detention and they’ve only been there for a short time, or they are 17 turning 18 and they are on the verge of missing out on school services…the disjunction comes when the state government has to provide the education and the Department of Immigration has to pay for it and if there is swaying over how much money its going to cost it just doesn’t happen.*—***Key Informant 2***

#### Health and well-being

All the participants reported that in WA they have better access to healthcare, with access to medical treatment if needed. Language is a barrier to this access, especially on arrival when English language proficiency is minimal. Two Afghani youth who were unaccompanied arrivals relied on guardians or friends for translation assistance. Interpreters were used by almost all the refugee youth at medical facilities while those that used an interpreter reported a better experience with the health care system.

Interestingly, the only emotional health issue mentioned was that of being torn between newfound freedom in Australia and the sadness that came from leaving behind friends and family. This was rather unusual as higher levels of mental health issues such as post-traumatic stress disorder (PTSD) and depression were possible given their history of trauma and dislocation and current circumstances of enforced separation from loved ones. However, these young refugees were also very resilient. Participants all commented on the unpleasant situation they had experienced in transient countries (living in refugee camps for years, limited access to education and health, people’s attitudes toward refugees) and reported that they felt emotionally better in Australia.

*“I like it (here) because it’s good for me to see the doctor. Because in Africa we were sick like 2* *years, 3* *years without seeing the doctor, so I don’t know if I’m sick or not. But here when I visit the doctor I can know all what is going. So for me, yes I like it.”*—***Congolese Female***

*“The positive thing is that the service here is better than my country, the negative thing is you miss your parents and sister and brothers.”*—***Iraqi Female***

*A medical appointment is important, so it is important to make sure they bring the paper work they need. And if they need somebody to be there for them because the majority of our clients are single adult males in their 20 or 30s and they are completely by themselves.*—***Key Informant 1***

#### Employment

The Congolese females stated that they were focusing on education rather than employment. The males were all looking for jobs (only two were employed at the time of the study) and reported finding it difficult to find employment. The three Afghan participants had vocational skills that were not recognized in Australia resulting in considerable disappointment. Participants reported having limited access to computers and the internet and not being familiar with these technologies as their main difficulty given that most job opportunities in Australia are posted online.

*“I want to study business management. My second plan is community services.”*—***Congolese Female***

### Social ecology themes

#### Social activities

Refugee youth participants reported limited social activities outside of structured support and their community. Most of their social interaction took place at the college. The Congolese males played soccer in a league and the Afghani men played sport at least once a week with other men from Afghanistan. The Congolese women’s social activities revolved around church events. The main social activity with their own community was through church or special functions, with Afghan and Congolese ethnic communities providing limited support to youth for everyday social life (going out with friends and family, church, sport).

*“There’s a place that everyone can play, Afghani guys. And if you like you can play soccer and if you like you can play basketball or volleyball.”*—***Afghani Male***

#### Religion

All of the youth from the DRC mentioned that they went to church at least three times a week. The Afghani youth also attended religious Islamic celebrations.

*“For me it’s three times a week, we have a church service in Wednesday night, Saturday night and Sunday morning.”*—***Congolese Male***

*“We always go to mosque to pray, I meet many Muslim sisters there and feel happy”*—***Iraqi Female***

Provision of free transport facilitated refugee youth attending church regularly. Almost all boys took part in sports which had helped them to find friends and socialize, while the girls didn’t play sports so lacked opportunities to meet others outside of school. Religion (Islam or Christianity) played an important part in the lives of the youth.

#### Support structures

All participants expressed that they received minimal assistance from caseworkers. The unaccompanied minors relied mostly on guardians for support or friends that were resettled in WA. Guardians were usually community members from the same ethnic group and for unaccompanied refugee youth aged 16–18 years, the state specifically the Department of Child Protection is their guardian and they are normally provided with a group home. Only one of the participants had an Australian friend and the rest had friends from school or other refugee background.

Those with family in Australia relied upon family members to discuss and solve problems, and two female participants mentioned receiving a small level of support from their neighbours. Those entering Australia on their own mostly received support from friends of the same ethnicity. One of the key informants mentioned a successful community volunteer program that community members could register and DIAC would supply them with the necessary services available to refugees.

*“Actually they (the caseworkers) should help us for 6*–*7* *months but they didn’t, they helped us just for 1* *month or 2* *months and after that they didn’t care about us. And it was very hard.”*—***Afghani Male***

*“I have one friend she has been here 10* *years. She is my best friend, when I have any problem I go to her and she says to me do this and that.”*—**Congolese Female**

*“The good thing I found here that was never in Sudan is that neighbours help a lot. Every time I want to go out, my neighbour asks me “do you know when the bus comes”. My neighbour is Australian and she is too nice.”*—***Sudanese Female***

*“Community volunteer program was discontinued and despite pushes for that to be reinstated* -*because it was a really successful program*- *nothing happened.”*—***Key Informant 2***

### Cultural capacity themes

#### Connection to family

Refugee youth felt they shouldered responsibility to provide financial and emotional support to the family remaining behind in their home country, and it was a big concern for both youth and their families. They constantly felt upset that their family members, siblings or parents were left behind in the country of conflict or transit country. They are able to contact and talk to family and do so often, sometimes unwittingly incurring high telephones bills when they first arrive in Australia

*(When asked if he felt sad) “Yes, absolutely, because my family in Afghanistan, they live there in danger and I can’t do anything”*—***Afghani Male***

*“It’s very difficult here. I came here alone. I had a big family there; I haven’t seen them in a long time now. I have to send money back home…They think I have lots of money here, but they don’t know the expenses here are very high.”*—***Pakistani Male***

*“I talked to mum and said I miss you mum I want to come back and live with you.”—****Sudanese Female***

#### Parental language and skills (reversal of cultural roles)

All participants who arrived with family found that their parents had more difficulties learning English and this increased their own responsibility as they had to provide communication support. Some participants also stated that their parents had lost the status they had in their home country due to their lack of proficiency in language. Many refugee youth had to help their parents with their everyday activities in Australia.

*“English is the number one need (discussing needs), so getting our clients to set on English programs whether it is to bringing clients to English classes or doing one*-*on*-*one assistance at home or doing it here (office) is really important.”*—***Key Informant 1***

*“My dad my mum they weren’t able to speak so I was the only one who can understand. So we went out together and I showed them…”*—***Congolese Female***

The youth were required to help whenever an interpreter was needed (such as for a doctor’s appointment).

*“[Sharing their experience of attending hospital] Maybe I think we should just [have] some interpreter for other languages that can’t speak English it’s very hard to go to the hospital.”*—***Afghani Male***

#### Aspirations for the future (reflects all themes)

All participants had plans for the future. They indicated a greater feeling of freedom and opportunity compared to their home country. For example, the Congolese girls shared plans to go into business management. The male participants planned to finish Technical and Further Education (vocational training) and to find a job as police officers, army officers, mechanics and others. Most indicated they felt like they had many more options for study and work in Australia.

*“In future I want to finish school. Go to another school or uni. I want to do my course then I can plan future to be nice to find a good job.”*—***Congolese Female***

*“Actually I am very happy. Because here I have peace and lot of opportunity for the future and I have freedom. I am free to do anything I want.” “I think Australian government is good, because if someone wants to become everything they can really.”*—***Afghani Male***

Overall the participants shared that they had a better life, peace and freedom in Australia despite unemployment, and were grateful they had been resettled in this country.

*“My older brother is in second country and we don’t have any opportunity to sponsor him. If a family or anyone came to Australia, the government should take it easier. It’s a lot of rules and time and money”*—***Afghani Male***

*“Everyone comes to Australia and they don’t have lot of money and Centrelink pays small money, just enough for food, rent and clothes.”*—***Afghani Male***

*“They have to learn English, 100% before coming to Australia. It’s like a key in your life here. If you have this key here you can open lot of doors.”*—***Iraqi Female***

#### Some recommendations shared by refugee youth

At the end of the FGD the refugee youth were asked if there had any recommendations for improving resettlement experiences. The Afghan youth expressed the desire to be able to sponsor family members to resettle in Australia. The timeframe of 7–13 years before family members can be resettled (according to DIAC’s policy) was described as disheartening. Overall, participants said that although support received through Centrelink was appreciated, it did not cover all expenses and they often had financial difficulties. One participant recommended more help in finding housing. Many participant mentioned learning English prior coming to Australia and ongoing English language support as the key to be successful.

## Discussion

This qualitative study drew on the experiences of a small number of refugee youth from Afghanistan and the DRC who have arrived on humanitarian visas in comparison to those who arrive by boat seeking asylum and examined factors that help or hinder successful resettlement. The analysis of both FGD and the key informant interviews revealed that resettlement experiences were strongly connected to language, education, social activities, support structures and health. The refugee youth experienced both positive and negative resettlement experiences during resettlement.

The strategies adopted by the refugee youth that enhance their coping and resettlement are learning the English language, and developing new skills (such as computer skills), engaging in sports, furthering their education to increase their employment opportunities, and seeking support from family and through religious activities. Many of these findings were consistent with the experiences of refugee youth identified in previous research internationally [4, 5, 8, 9, 13].

A relevant and recurring theme found was the issue of language proficiency, either when accessing health, employment and commercial services or as a barrier to study and work in the new country. Previous studies have documented that higher English language proficiency increases confidence in accessing services [5, 28]. Despite the fact that refugee youth appreciated the free medical treatment available in Australia, lack of English proficiency and interpreters in the health system was a frustrating barrier.

To our knowledge, this is one of few studies that documents the impact of the lack of recognition of prior education and learning on refugee youth resettlement. Some of the older youth who had some skills in their home or transient countries could not have these recognised in Australia. All of our participants reported a positive education experience with teachers willing to help refugee students from a refugee background [14]. Social activities and support networks in this group revolved around family, friends and religious groups with similar background [9, 10, 13], highlighting the importance of culture and religion but also how these aspects influence the social ecology.

Limited support from caseworkers was an important finding and is an area that warrants further exploration. It is possible that his may be a reflection of the large proportion of humanitarian entrants who are IMAs and the punitive approach taken by the Australian government which has meant community support workers to assist with resettlement are under resourced and there is a greater dependence on volunteers.

A suggestion shared by the key informants in this study was that community volunteer programmes could greatly assist the resettlement of unaccompanied minors and IMAs. Despite continuing change in government policy, actions can be taken by migration support organisations to facilitate successful resettlement [1, 11, 29]. Emphasis on engaging community members, providing more assistance in the first year of resettlement, education on available language and health resources will aid the process of successful resettlement. Community volunteer programs provide access to government services, interpreters, legal advice, localised help sources and also help with integration. Previous studies also support the recommendations shared by the participants and key informants of enhancing English language skills, implementing strategies to improve educational outcomes and providing greater community support [14, 30, 31].

Thus, recommendations proposed by refugee youth and key informants need to be taken into consideration to build resilient communities of refugee youth with a strong capacity to cope and thrive. There are severe long term impacts on refugees who are unable to gain English proficiency in terms of their ability to work and social connections which highlight the critical importance of early support to assist refugees with adapting and integrating in their new country [32]. The problems with resettlement that were identified in older refugee youth, many of whom had been in Australia for many years, reflect that their aspirations for resettlement had not been realized [33].

The study provided rich data from a small group of participants however there are a few limitations to the study. The sample was small and thus whilst not possible to generalise the findings, the recommendations proposed are transferable and add to the growing body of research on refugee youth. The funding constraints meant that the study could not be expanded to include more participants.

This capacity to cope and be resilient may be adversely affected with more restrictive government policies that were implemented in July 2013. The Australian Government implemented new policies pertaining to those arriving by boat, stipulating detention and processing in centres in Nauru or Papua New Guinea and no resettlement in Australia [34]. In September 2013, there was further policy change and IMAs were referred to as illegal maritime arrivals and a media embargo on reporting was implemented, changes which are considered a violation of international human rights law [35]. There is already evidence of the adverse impact of these punitive approaches on the mental health of refugees, but what is often not recognised is the adverse impact for those who are trying to resettle in Australia when their families remain in danger elsewhere. In 2014, there was a name change from the DIAC to Department of Immigration and Boarder Protection (DIBP). Although these changes did not directly impact the study participants these changes had impacts for family members remaining behind and meant that they possibly could not be resettled in Australia. These changes also impact wider public perception and how refugees are viewed by society [36].

## Conclusion

The results of this study highlight that refugee youth participants are resilient, want to succeed and have aspirations for the future. This study drew on the psychosocial framework and considered the culture, values, and human capacity of the youth. Our findings support previous Australian and international literature which highlight that programmes promoting a sense of belonging and those that promote coping and adaptation are vital and much needed for the well-being of refugee youth. The hostility portrayed by the current government towards asylum seekers allows a humanitarian issue such as refugee resettlement and asylum seeking to be translated into a security and border protection issue, resulting in refugees and asylum seekers being perceived as a threat. Refugee youth already in Australia need to be provided with the opportunity to thrive, to be included and to belong. Their efforts to forge a new identity in their resettled country, is often shaped by prevailing social and government policies. There is an urgent need for a more humane approach that ensures sustained opportunities for education, skill enhancement, and inclusive policies that allow refugee youth to become resilient and independent future citizens in multicultural Australia.

## Abbreviations

DIAC: Department of Immigration and Citizenship
DRC: Democratic Republic of Congo
FGD: focus group discussion
IMAs: irregular maritime arrivals (used by government before August 2013), illegal maritime arrivals (used by government before August 2013)
PTSD: post-traumatic stress disorder
WA: Western Australia
